# Epidemiology and outcome of cardiac arrest in patients with liver cirrhosis

**DOI:** 10.1186/2197-425X-3-S1-A687

**Published:** 2015-10-01

**Authors:** K Roedl, K Rutter, T Horvatits, A Drolz, H Herkner, F Sterz, V Fuhrmann

**Affiliations:** Intensive Care Medicine, University Medical Center Hamburg-Eppendorf, Hamburg, Germany; Department of Emergency Medicine, Medical University of Vienna, Vienna, Austria

## Introduction

Sudden cardiac arrest (CA) is one of the leading causes of death in adults in many parts of the world [1]. Every year estimated 350.000 to 700.000 people in Europe are suffering CA and receive cardiopulmonary resuscitation (CPR) [2]. To date, there is no data available on CA and CPR in patients with liver cirrhosis.

## Objectives

Aim of the study was to determine cause and outcome of CA in patients with liver cirrhosis after CPR.

## Methods

Out of a cohort of 1068 consecutive patients who were successfully resuscitated and treated at the Medical University of Vienna, 47 (4%) patients with liver cirrhosis could be identified. Patient characteristics, admission diagnosis, severity of disease, course of the disease and 28d mortality were assessed.

## Results

Overall 47 patients (35 male, age 60 ± 11 years) with liver cirrhosis and CA with successful CPR were assessed. 17 patients had Child-Pugh A, 17 Child-Pugh B and 13 Child-Pugh C. SOFA-Score on admission was 11 ± 5, MELD-Score was 18 ± 8 on admission. Cause of liver cirrhosis was alcohol in 35 patients, viral hepatitis in 6 patients and other causes in 6 patients.

Cardiac events leading to CA were observed in 21 (44%) patients. Initial rhythm was VF/VT in 10 (21%) patients, asystole in 11 (24%) patients, PEA in 24 (51%) patients and unknown in 2 (4%) patients. 31 (66%) patients suffered CA out-of-hospital. Time to ROSC was 17 ± 15 minutes.

28d-survival was overall 28%. 37 (78%) patients were dead after 1 month or had bad neurological outcome (CPC III/IV). None of the patients with Child-Pugh C cirrhosis survived 1 month with good neurologic outcome.

## Conclusions

Cause of CA in patients with liver cirrhosis is rarely cardiac. About one quarter of patients with liver cirrhosis survived more than 28d after successful CPR. No patient with Child-Pugh C cirrhosis survived longer than 28d after successful CPR with good neurological outcome.
